# Innovations in aging biology: highlights from the ARDD emerging science & technologies workshop

**DOI:** 10.1038/s41514-025-00193-5

**Published:** 2025-02-18

**Authors:** Maximilian Unfried, Tomas Schmauck-Medina, Neal D. Amin, Edward S. Boyden, Georg Fuellen, Jing-Dong Jackie Han, Jacob H. Hanna, Indra Heckenbach, Konstantin Khodosevich, Lisa Melton, Emad Moeendarbary, Tae Seok Moon, Shahaf Peleg, Anders Sandberg, Lingyan Shi, Daniela Bakula, Alex Zhavoronkov, Morten Scheibye-Knudsen

**Affiliations:** 1 Healthy Longevity Translational Research Program, Yong Loo Lin School of Medicine, National University of Singapore, Singapore, Singapore; 2 Department of Biochemistry, Yong Loo Lin School of Medicine, National University of Singapore, Singapore, Singapore; 3 Department of Clinical Molecular Biology, University of Oslo and Akershus University Hospital, Lørenskog, Norway; 4 Department of Psychiatry and Behavioral Sciences, Stanford University, Stanford, CA 94305 USA; 5 Departments of Brain and Cognitive Sciences, Media Arts and Sciences, and Biological Engineering, K. Lisa Yang Center for Bionics and McGovern Institute, MIT, Cambridge, MA USA; 6 Koch Institute, Massachusetts Institute of Technology, Cambridge, MA USA; 7 Howard Hughes Medical Institute, Cambridge, MA USA; 8 Rostock University Medical Center, Institute for Biostatistics and Informatics in Medicine and Aging Research (IBIMA), Rostock, Germany; 9 UCD Conway Institute of Biomolecular and Biomedical Research, School of Medicine, University College Dublin, Dublin, Ireland; 10 Peking-Tsinghua Center for Life Sciences, Academy for Advanced Interdisciplinary Studies, Center for Quantitative Biology, Peking University, Beijing, China; 11 Peking University Chengdu Academy for Advanced Interdisciplinary Biotechnologies, Chengdu, China; 12 International Center for Aging and Cancer, Hainan Medical University, Haikou, China; 13 Department of Molecular Genetics, Weizmann Institute of Science, Rehovot, Israel; 14 Center for Healthy Aging, Department of Cellular and Molecular Medicine, University of Copenhagen, Copenhagen, Denmark; 15 Buck Institute for Research on Aging, Novato, CA USA; 16 Biotech Research and Innovation Centre, Faculty of Health, University of Copenhagen, Copenhagen, Denmark; 17 Nature Biotechnology, Springer Nature, London, UK; 18 Department of Mechanical Engineering, University College London, London, UK; 19 199 Biotechnologies Ltd, London, UK; 20 Synthetic Biology Group, J. Craig Venter Institute, La Jolla, CA USA; 21 Research Institute for Farm Animal Biology (FBN), Wilhelm-Stahl-Allee 2, 18196 Dummerstorf, Germany; 22 Institute for Futures Studies, 101 31 Stockholm, Sweden; 23 Shu Chien-Gene Lay Dept. of Bioengineering, University of California San Diego, La Jolla, CA 92093 USA; 24 Insilico Medicine US Inc, 1000 Massachusetts Avenue, Suite 126, Cambridge, MA 02138 USA

**Keywords:** Experimental organisms, Metabolomics, Biological techniques, Developmental biology, Drug discovery, Molecular biology, Neuroscience, Stem cells, Biomarkers, Diseases, Medical research

## Abstract

The field of biogerontology has established itself through significant lines of research in recent decades. However, despite early breakthroughs, progress in understanding the aging process has been slow. To push the field forward, new methodologies and technologies are likely needed to unravel the complexity of aging. This meeting brought together leading scientists and innovators to explore some emerging approaches, presenting groundbreaking advancements in four key sessions, culminating in a panel discussion.

## AI advances in aging biology

The first session focused on the use of artificial intelligence to advance our understanding of complex biological phenomena. Jackie Han from Peking University presented her latest work using their 3D facial map technology to predict age^1^ in two distinct populations: one from Ghana and another from China. Her findings revealed that while both populations age similarly along the depth axis of the 3D face, the African population exhibited slower aging on their ethnic-shared 3D facial aging clock. Although Ghana’s average life expectancy is lower than that of China, her group found African-likeness immune-associated processes in Asians may alter facial appearance in a youthful-like way, for example the face looks tighter and more lifted. Finally, they found that genes associated with African-like features were linked to immune processes, such as neutrophil degranulation. Her group is now using this technology to study phenotypic biomarkers of aging to a broader understanding of human aging and its variability.

With regard to the hallmarks of aging, a problem that has been present in the field is the lack of specific biomarkers for senescent cells. Indra Heckenbach, from the University of Copenhagen, addressed this challenge through the use of deep learning to discriminate senescent cells based on nuclear morphology^2^. A surprising application of this method was used on breast tissue to predict cancer. The group found that two of their trained models significantly correlated with the post-diagnosis of breast cancer, and when one of these models was combined with the Gail-score they were able to find a stronger odds ratio^3^. The group as well found that through their model it was also possible to predict risk of development of cancer from benign breast disease.

Finally, with the rise of tools such as large language models (LLMs), Georg Fuellen from the Rostock University Medical Center in collaboration with Brian Kennedy from the National University of Singapore, presented novel insights on the use of these tools for creating AI-based longevity recommendations^4^. The model needs to be effective at evaluating the interventions with an understanding of the patient (or the healthy subject asking for recommendations) in mind. Thus, the subject needs to provide a biomarker profile^5,6^, which in turn must be considered appropriately by the LLM. In addition, the use of LLMs for designing effective longevity studies was discussed. In particular, asking GPT4o for longevity study designs, it returns standard designs centering around diet and exercise, as these best reflect the “short head” of the training data distribution. However, with some nudging, designs for, e.g., testing epigenetic rejuvenation interventions can be obtained. It may be worthwhile to start a “Leaderboard” of good designs, contributed and evaluated by humans and LLMs alike. This work emphasizes the importance of tailoring interventions based on patient profiles, as well as considering key factors such as study population and feasibility when designing longevity studies.

## Brain aging and neurotechnology

As our knowledge in neuroscience deepens, cutting-edge technologies and ideas are providing new avenues to address neurodegenerative diseases and aging itself. This session delved into a range of innovative approaches aimed at preserving and even enhancing brain function in the face of aging.

Anders Sandberg, from the Institute for Future Studies, Stockholm, provided a thought-provoking analysis of the technological and philosophical implications of “mind uploading”, a term that Sandberg expressed his reservations about since it presupposes much about the nature of the mind (whole brain emulation is more accurate). He outlined several assumptions necessary for its realization, such as the dependency on physical processes, computational demands, and the existence of scale-separation^7^. One particularly interesting assumption he made was the one of “brain-centeredness,” where a full-body simulation of the same resolution as the brain is not essential for the success of mental emulation. The resolution (in space, time and complexity) of the emulation does not have to be the same everywhere. Sandberg referenced major technological milestones from the past two decades, including the Blue Brain project and advancements like expansion microscopy (ExM). While computational power and scanning technologies have seen significant progress, Sandberg suggested that the real challenge lies in integrating these developments into a comprehensive and structured pipeline. The discussion that followed his presentation ventured into philosophical territory, suggesting that these scientific advancements might even help address age-old philosophical questions about consciousness and identity.

Neal Amin, from Stanford University, presented a different approach focusing on neural replacement therapy to combat age-related brain pathologies. He emphasized that as neurons are lost in diseases such as Parkinson’s, replacing them becomes essential for treatment. Amin highlighted clinical trials such as ASPIRO and STEM-PD, which are investigating neuron replacement in Parkinson’s Disease patients. However, a major challenge in this field lies in the brain’s vast cellular diversity, despite the fact that relatively few morphogens guide the differentiation of these cell types. To overcome this, Amin has designed a pipeline that optimizes key parameters such as timing, concentration, and duration of morphogen exposure to produce specific cell types^8^. This tool not only enhances our understanding of brain development, pathology, and the aging process, but also holds promise for improving organoid generation and cell transplantation therapies.

Concluding the session, Konstantin Khodosevich from the University of Copenhagen tackled the complex challenges of psychiatric disorders, particularly how mental health issues can arise from various risk factors during brain development and maturation. His team’s research into single-nucleus RNA sequencing of the cortex from schizophrenia patients revealed a previously uncharted network of transcription factors^9^. He proposed that spatial transcriptomics, which maps gene expression patterns within tissue, could become a key tool in unraveling the intricate changes occurring in the aging brain, potentially offering new insights not only into mental health but as well in the cognitive changes that are specific to the process of aging. Finally, he proposed how large-scale implementation of single-cell and spatial data can help to improve drug target identification and preclinical studies^10^.

## Emerging technologies I: cryopreservation, mechanobiology and ex vivo organ development

As the field of biogerontology expands, a wide array of emerging technologies is being explored to tackle the challenges posed by aging and its associated biological limitations. This session was highlighted by pioneering novel approaches that could reshape how we think about organ preservation, tissue mechanics, and regenerative medicine.

João Pedro de Magalhães, from the University of Birmingham, opened the session by addressing the critical challenges in organ transplantation, highlighting that only 30% of transplantable hearts and 20% of lungs are actually utilized^11^. He highlighted cryopreservation as a potential solution. While embryos can be frozen for decades, the cryopreservation of larger organs remains elusive, not to mention entire bodies. De Magalhães emphasized the role of artificial intelligence in discovering new cryoprotective agents, which could lead to breakthroughs in cryopreservation. This has far-reaching implications in fields like reproductive medicine, organ banking, space travel, and even human medical biostasis^12^. Interestingly, de Magalhães noted that the problem of cryopreservation is more straightforward than that of aging itself, as the challenges are clearer and better understood, whereas in aging, there is not even consensus on how to spell it (aging/ageing)^13,14^.

As organisms age, their cells experience mechanical changes that can affect protein structure, cell migration, tissue integrity, and vascular stiffness. Emad Moeendarbary, from University College London, described these changes and how they can be tracked using techniques such as traction force microscopy and atomic force microscopy (AFM). In neurology, AFM has been employed to obtain high-resolution images of the central nervous system’s tissue mechanics^15^. For example, Moeendarbary’s work has demonstrated that in Alzheimer’s disease models, certain brain regions experience a softening of the tissue^16^. Additionally, he explored the use of “organ-on-a-chip” systems, which allow for the study of 3D cell-cell interactions and incorporation of chemokine and flow gradients. As alternatives to animal models, these systems could prove instrumental in further understanding the biomechanical and structural changes that organs undergo with age.

Jacob Hanna, from the Weizmann Institute of Science, presented a promising approach for regenerative medicine. In previous studies, Hanna demonstrated how sickle cell anemia could be corrected by extracting skin cells from a mouse, reprogramming them to correct the genetic mutation, and then reimplanting them as blood stem cells^17^. However, Hanna discussed a key challenge in this process: cells extracted from a patient, which are pluripotent, are differentiated in vitro into advanced embryo models. This process bypasses the blastocyst stage, thereby alleviating potential ethical concerns about the illegal implantation of such models, as post-blastocyst entities from any mammal can never implant into the uterus. He argued that in order to achieve proper in vitro reconstitution for cell replacement therapies, cells need to be cultured from a naïve state and then differentiated into embryoid-models using an ex utero culture device. DNA methylation is reset in naive pluripotency, hence the DNA based “aging epigenetic clock” is reset in naive pluripotent cells. The in house developed device is a sophisticated incubator system that is conducive for advanced embryo-like growth outside the womb, as it controls parameters like pressure, temperature, oxygen, nutrients, etc. The device is in constant rotation to avoid unwanted attachment of developing embryo models and presents different solutions for different developmental stages^18^. Recently, using this method it has been possible to model the key developmental stages of gastrulation, neurulation and organogenesis, reaching beating heart-like structures, a gut tube and brain regions in mice^19^. This could pave the way for more effective cell therapies and ex vivo organ development in the future, specifically targeting the issues with regards to organ transplantation.

## Emerging technologies II: tooling and synthetic biology approaches

The final session of the meeting delved into advanced technological innovations that promise to transform our understanding of, and control of, biological systems. First Ed Boyden, from MIT, opened the session by identifying three key challenges in acquiring ground truth-oriented data for accurate computer modeling of biological systems: (1) mapping molecules and their interactions, (2) controlling high-speed signaling dynamics, and (3) observing these dynamics. To tackle the first challenge, Boyden discussed the use of ExM, which allows biological samples to be evenly expanded, thereby enhancing resolution down to the nanoscale, and enabling detailed 3D reconstructions of cells and tissues^20^. For controlling high-speed dynamics, he highlighted optogenetic technology, where transgenes can be used for the temporally precise optical activation of specific pathways, such as the electrical activity of targeted neurons. Lastly, to observe these complex dynamics, Boyden introduced a novel fluorescent reporter system utilizing self-assembling peptides to form clusters, which can monitor different signals at different points within a living cell^21^. He also presented a method that distinguishes simultaneous signals by associating different signals with fluorophores of different switching speeds, allowing mathematical unmixing of recorded data into individual signals^22^. These tools could potentially be unified to collect data to support the simulation of biological systems.

Lingyan Shi, from the University of California, San Diego, followed by discussing the integration of three types of microscopy techniques to study metabolism. Shi identified three main challenges: lack of molecular specificity, the need for super-resolution imaging, and the ability to differentiate between newly synthesized and pre-existing molecules. She demonstrated how each challenge could be addressed through advanced microscopy. First, by using Raman spectroscopy, she explained how unique molecular profiles could be detected in a label-free manner, such as tracking cholesterol^23^. For improving resolution, she applied A-PoD microscopy, which utilizes fluorescent switches to optimize visualization^24^. Finally, Shi introduced a bioorthogonal label-free stimulated Raman scattering (SRS) microscope that can differentiate between new and pre-existing molecules, which may allow researchers to follow key biological processes like autophagy, a critical function in aging^25^.

Tae Seok Moon, from the J. Craig Venter Institute, presented an innovative approach to combat dysbiosis, a hallmark of aging, by genetically engineering the gut microbiome. His team developed a strain-specific, CRISPR-guided, RNA-based bacterial killing technology to target specific microbes with high precision^26^. This approach allows for targeted killing of specific bacteria in the gut by selecting and controlling the right microbial strains for gut health. Moon noted that treating different aging-related diseases might require different microbial profiles, so optimizing these interventions will be key to their success in the clinic.

Finally, Shahaf Peleg, from FBN Dummerstorf, stated that our biological lifespan may be limited by inherent constraints, and that the only way to significantly extend it might be through synthetic redesign. Peleg discussed the concept of “animal models 2.0”, genetically modified upgraded animals for the purpose of aging studies. For example, he discussed modifying light activated proton pumps such as from Leptosphaeria maculans (Mac) or Bacteriorhodopsin (Br) and fusing them with mammalian targeting sequence mitochondria and mammalian transmembrane domains in order to ultimately repurpose their function as part of ATP synthesis^27^, with his work focusing on integrating these novel systems using optogenetics for energy transduction^28^. Further, he discussed the idea of using genes from other organisms, such as plants, to combat problems like protein aggregation in animals. He emphasized that such interventions could revolutionize biology, but significant challenges remain, including how to control the expression of synthetic genes over time and how to ensure the optimal presence of such proteins in the body.

## Conclusion

The Emerging Science & Technologies Workshop at ARDD 2024 provided a unique platform for exploring unconventional approaches to solving biological challenges. The workshop concluded with a panel discussion moderated by Lisa Melton from *Nature Biotechnology*, featuring Lingyan Shi, Tae Seok Moon, Ed Boyden, and Jacob Hanna. The panel underscored the need to focus on the dynamic and interconnected nature of metabolic systems, moving away from linear “A to B to C” models and towards a more systematic understanding of biological processes.

Additionally, the discussion highlighted the underutilization of existing technologies and resources, with panelists stressing the importance of increasing awareness about what is technologically possible. For instance, a surprising gap in our knowledge is the lack of complete genome sequences for many animals, which could significantly advance research if addressed. Finally, the panel pointed to the future of high-throughput systems and the emerging field of single-protein sequencing, both of which were seen as transformative tools that could revolutionize our ability to understand and manipulate biological systems.

## Data Availability

No datasets were generated or analysed during the current study.
